# Plasminogen Activator Inhibitor-1: Another String in the Platelet-Derived Extracellular Vesicles Bow?∗

**DOI:** 10.1016/j.jacbts.2022.06.016

**Published:** 2022-10-24

**Authors:** Dimitri Arangalage, Gregory Franck, Giuseppina Caligiuri

**Affiliations:** a Cardiology Department, AP-HP, Bichat Hospital, Paris, France; b INSERM U1148, Université Paris Cité, Paris, France

**Keywords:** biomarkers, coronary artery disease, extracellular vesicles, plasminogen activator inhibitor-1

**Figure:**
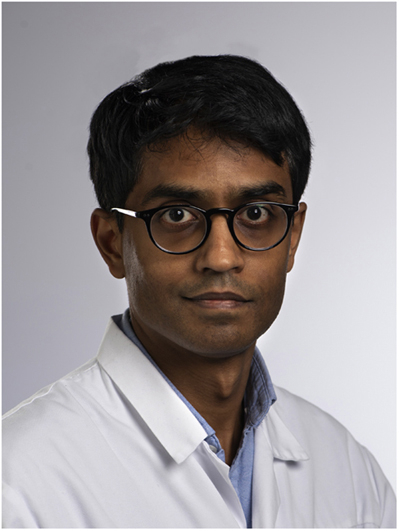

Beyond traditional risk factors and despite significant advances in percutaneous coronary intervention techniques and pharmacologic therapies for atherosclerosis, a “residual” risk still burdens the outcome of patients with coronary artery disease. The quest for a reliable biomarker capable of independently predicting the occurrence of coronary events has been ongoing for decades, but meaningful biomarkers applicable in clinical routines have proved to be particularly elusive. Indeed, despite extended experimental research activity in the past 30 years, the pathophysiology of atherosclerosis remains only partially understood. Identifying pathways and molecular triggers that contribute in the clinical condition to the increase of the residual risk of atherosclerotic disease may not only lead to the discovery of clinically relevant biomarkers, but also point toward novel potential therapeutic targets.

In this issue of *JACC: Basic to Translational Science*, Jung et al^1^ elegantly embraced such an objective and demonstrated, by combining in vitro experiments and a population-based study, that platelet-derived extracellular vesicles (EVs) expressing plasminogen activator inhibitor-1 (PAI-1^+^ PEV) may represent a promising predictive biomarker for major adverse cardiovascular events (MACE) and constitute a potential therapeutic target. A striking association between high PAI-1^+^ PEV levels and MACE (HR: 2.43; 95% CI: 1.43-4.13; *P* = 0.001), even after adjusting for recognized clinical parameters, is documented in this study. Of note, PAI-1^+^ PEV levels significantly improved the MACE prediction model when combined with age, diabetes, and acute coronary syndrome. It is evidently essential to replicate these results in larger cohorts of patients in prospective and multicenter studies to confirm the validity of this biomarker. It is also important to bear in mind that for the time being, the wide use of PAI-1^+^ PEV as a biomarker remains limited by the technical difficulty to implement a routine dosage.

EVs are a heterogeneous group of lipid bilayer–delimited particles released from the cellular plasma membrane or endosomal compartment of a variety of cells. The main feature of EVs is their ability to transport diverse cargo, including RNA, lipids, and proteins, which may be transferred to cellular recipients. Therefore, EVs are recognized as major mediators of intercellular communication. PEVs are released by platelets on activation and have been linked to various conditions including atherosclerosis and coronary artery disease. Jung et al^1^ demonstrated that a consistent fraction of PEVs carries PAI-1, an enzymatic product of cardiovascular tissues critically involved in atherothrombotic events. The release in the peripheral blood of patients at risk of PAI-1^+^ PEV complexes, documented with the use of both flow cytometry and electron beam microscopy, holds a double value: It is a clinically relevant biomarker because it reflects the extent of ongoing platelet injury at sites of atherothrombosis, and it represents a putative critical therapeutic target, because PAI-1^+^ PEV complexes may directly exacerbate the risk of MACE by triggering further pathologic responses by local endothelial and smooth muscle cells (SMCs).^1^

PAI-1 is a member of the serine protease inhibitor (serpin) family and was historically found to participate to the regulation of fibrinolysis through the inhibition of tissue plasminogen activator and urokinase-type plasminogen activator. Consequently, one may intuitively argue that the pathophysiologic mechanism leading to coronary events mainly relies on the prothrombotic effect of PAI-1 through the prevention from fibrinolysis of the developing thrombi at the site of atherosclerotic plaque rupture or endothelial injury induced by stent expansion. The present study pleads in favor of a different mechanism, because incubation with PEVs enhanced significantly SMC migration and proliferation and promoted SMC phenotypic switching, and, most importantly, thrombogenicity was not affected in vitro by the proportion of PAI-1^+^ PEV, suggesting that the clinical end point in terms of MACE was essentially driven by the modulation of SMC biology (Figure 1). This observation is in line with past in vivo and in vitro studies that suggested that the influence of PEVs on hemostasis may be limited, and it strengthens the increasing evidence supporting the involvement of PAI-1 in vascular remodeling. However, the exact mechanism influencing arterial wound healing and neointima formation, particularly via regulation of SMC migration, remains controversial. On the one hand, PAI-1 has been shown to negatively affect SMC migration by inhibiting plasmin generation, which is involved in wound healing through activation of growth factors and proteolysis of extracellular matrix (ECM) constituents, and by blocking ECM vitronectin-binding interaction with αVβ3 integrin.^2^ On the other hand, PAI-1 may promote SMC migration through the activation of the Janus kinase/signal transducer and activator of transcription pathway following its binding to low-density lipoprotein receptor–related protein 1 (LRP1).^3^

**Figure 1:**
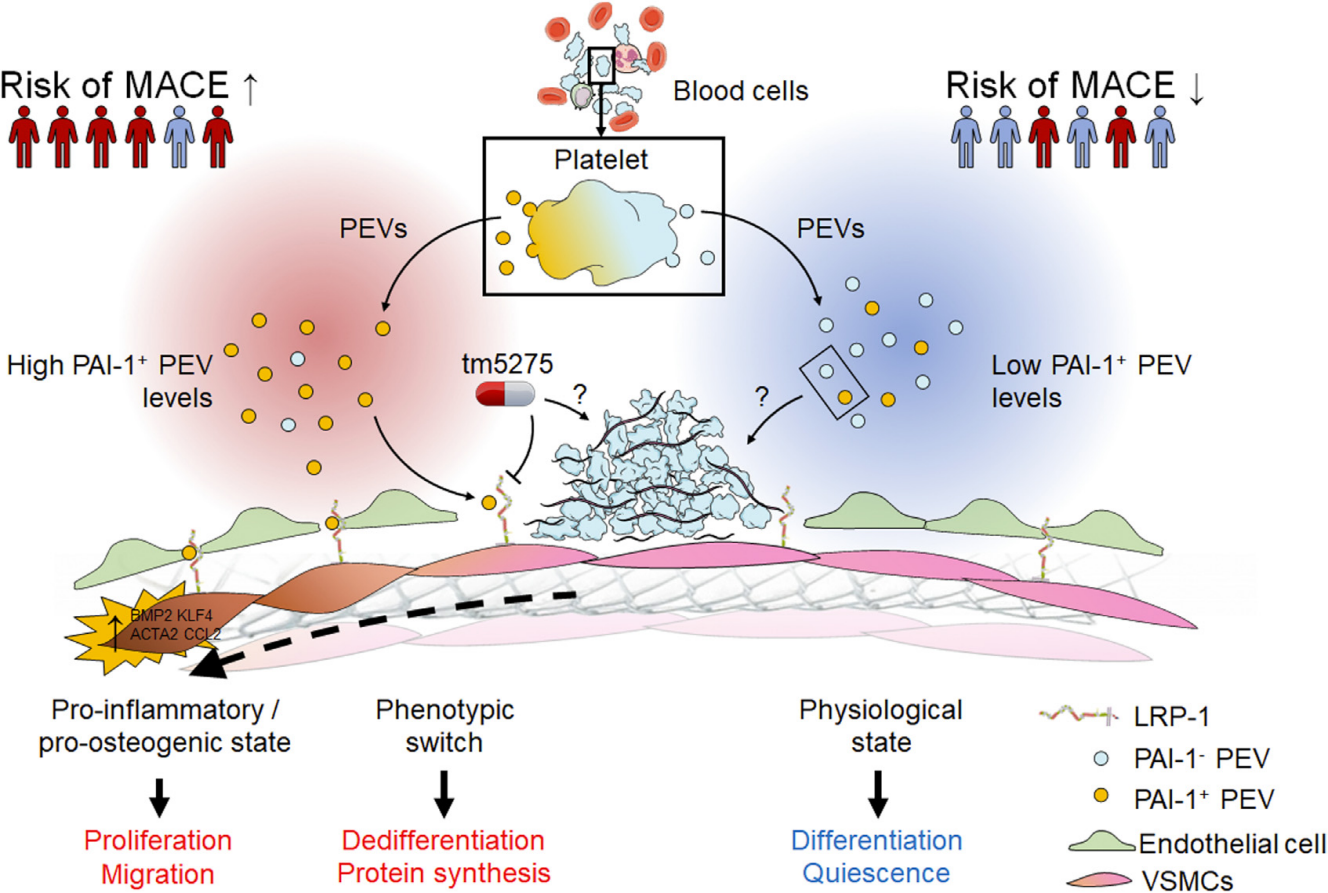
The Potential Role of PAI-1^+^ PEV in Coronary Artery Disease Platelet-derived extracellular vesicles expressing plasminogen activator inhibitor-1 (PAI-1^+^ PEV) promote smooth muscle cell (SMC) phenotypic switching toward a proinflammatory and procalcifying phenotype via the activation of the lipoprotein receptor–related protein 1 (LRP1) receptor. High PAI-1^+^ PEV levels were predictive of major adverse cardiovascular events (MACE). Inhibition of the PAI-1/LRP1 pathway with TM5275 dampened SMC phenotypic switching. Neither PAI-1^+^ PEV levels nor TM5275 affected thrombogenicity, but the exact mechanisms remain to be deciphered. VSMC = vascular smooth muscle cell.

An interesting finding of the present study is that TM5275, an orally bioavailable small molecule that inhibits the interaction of PAI-1 with LRP1, significantly reduced vascular SMC proliferation regardless of the PEV total amount. This observation certainly adds to previous findings in which the inhibition of PAI-1 similarly reduced SMC migration and prevented neointimal formation after vascular injury in mouse models,^4^ but caution is required when postulating that pharmacologic intervention to modulate PAI-1 would prove effective in humans as data in the literature are still preliminary. Considering that PEVs, along with the many other blood elements present in the circulation, carry a multitude of potentially pathogenic components, one may argue that inhibiting a single pathway would have a limited effect on processes taking place in the subendothelial space, where many complex physiologic pathways are triggered in pathologic conditions. Nonetheless, the pathway proposed by the authors holds a clear translational potential and warrants further interventional studies in preclinical models, aimed at providing the proof of concept in support of potentially relevant therapeutic applications.

The reach of the PAI-1^+^ PEV particles documented by Jung et al^1^ may go beyond the scope of coronary artery disease and extend to patients affected by valvular heart disease, a condition associated with endothelial injury at the site of pathogenic calcification, similarly to mechanisms observed in atherosclerosis.^5^ All sorts of blood elements can enter the subendothelial space through such subclinical intimal flaps and may compromise the local tissue repair process to eventually trigger a phenotypic transition of the stromal valvular cells toward a procalcifying osteogenic phenotype, ultimately leading to calcific aortic valve stenosis. Interestingly, the phenotypic transition of SMCs cocultured with PEVs toward a procalcifying osteogenic phenotype described by Jung et al and supported by the expression of bone morphogenic protein 2 is typically observed in transitioned valvular interstitial cells of diseased aortic valves.

In summary, the study by Jung et al^1^ points toward a potential pathogenic mechanism independent from classic risk factors and may contribute to match the still unmet residual risk of acute events in patients affected by coronary atherosclerosis or other cardiovascular conditions generated by similar cell biology transition and pathogenic features.

## Funding Support and Author Disclosures

The authors have reported that they have no relationships relevant to the contents of this paper to disclose.
